# Affordable Fabrication of Conductive Electrodes and Dielectric Films for a Paper-Based Digital Microfluidic Chip

**DOI:** 10.3390/mi10020109

**Published:** 2019-02-07

**Authors:** Veasna Soum, Yunpyo Kim, Sooyong Park, Mary Chuong, Soo Ryeon Ryu, Sang Ho Lee, Georgi Tanev, Jan Madsen, Oh-Sun Kwon, Kwanwoo Shin

**Affiliations:** 1 Department of Chemistry, Institute of Biological Interfaces, Sogang University, Seoul 04107, Korea; veasna_soum@yahoo.com (V.S.); hhh0411hhh@naver.com (Y.K.); qkrtndyd111@naver.com (S.P.); marychuong05@gmail.com (M.C.); navio23@sogang.ac.kr (S.R.R.); 2 Department of Chemical Engineering, The Cooper Union for Advancement of Science and Art, New York, NY 10003, USA; private.shlee@gmail.com; 3 Department of Applied Mathematics and Computer Science, Technical University of Denmark, 2800 Lyngby, Denmark; g.p.tanev@gmail.com (G.T.); jama@dtu.dk (J.M.)

**Keywords:** dielectric film, plastic wrap, ballpoint pen printing, conductive electrode, digital microfluidic chip, electrowetting

## Abstract

In order to fabricate a digital microfluidic (DMF) chip, which requires a patterned array of electrodes coated with a dielectric film, we explored two simple methods: Ballpoint pen printing to generate the electrodes, and wrapping of a dielectric plastic film to coat the electrodes. For precise and programmable printing of the patterned electrodes, we used a digital plotter with a ballpoint pen filled with a silver nanoparticle (AgNP) ink. Instead of using conventional material deposition methods, such as chemical vapor deposition, printing, and spin coating, for fabricating the thin dielectric layer, we used a simple method in which we prepared a thin dielectric layer using pre-made linear, low-density polyethylene (LLDPE) plastic (17-μm thick) by simple wrapping. We then sealed it tightly with thin silicone oil layers so that it could be used as a DMF chip. Such a treated dielectric layer showed good electrowetting performance for a sessile drop without contact angle hysteresis under an applied voltage of less than 170 V. By using this straightforward fabrication method, we quickly and affordably fabricated a paper-based DMF chip and demonstrated the digital electrofluidic actuation and manipulation of drops.

## 1. Introduction

A few decades ago, a digital microfluidic (DMF) chip was introduced as a new type of lab-on-a-chip (LOC) device [1,2,3]. The DMF chip manipulates droplets on the surface of a set of electrode arrays coated with a dielectric film and actuated by applying an electrical potential. The actuation principle is based on the so-called electrowetting on dielectric (EWOD) phenomena. EWOD is a very practical way for fluidic manipulation in microfluidic devices. It has been used for creating an electrowetting valve to control the flow of the fluid in a continuous-flow paper-based microfluidic device [4,5]. Moreover, EWOD has been effectively used for the mixing, splitting, and transporting of aqueous samples, which are essential characteristics for LOCs [6,7,8]. That type of LOC was a simplified and minimized device, because many scalable components, such as complex pumps, guiding channels and valves, had been removed and replaced with planar structures capable of actuating a DMF drop driven by the EWOD phenomena [9,10,11].

For the fabrication of a planar DMF chip, a set of patterned electrode arrays and a thin dielectric layer must be fabricated [1,2,3]. Many deposition methods can be used to pattern electrodes, but most rely on the phases of the deposited materials, such as metals with lithography and sputtering, wet-based inks with inkjet printing, copper-based printed circuit boards with etching, and so on [9,12,13]. Particularly, a paper-based DMF chip, which is much easier to implement for inkjet printing of patterned electrodes than any other conventional substrates, such as wafers, glasses and polychlorinated biphenyls (PCBs), was introduced, providing an easy and convenient means of fabricating DMF LOCs [8,14,15,16,17]. Commonly, thin dielectric layers for DMF chips are generated by using spin coating and chemical vapor deposition (CVD), which require a clean room facility [18,19]. These processes cause the fabrication of DMF chips to be expensive, even though the cost of LOC devices for point-of-care (POC) applications should be inexpensive.

Here we introduce and exploit a simplified way of printing that uses a ballpoint pen filled with ink made of a conductive material, as well as a digital plotter for printing electrode arrays on paper for a DMF chip. Moreover, even though parylene-C has been widely used for the deposition of dielectric films, especially when the CVD method is used, we attempted to devise a significantly simpler way to fabricate dielectric films; to that end, we explored a wrapping method that uses a pre-made plastic film. By combining the above two simplified deposition methods to deposit a double-layer, i.e., a dielectric layer top-coated onto a conductive layer, we affordably fabricated a paper-based DMF chip and successfully demonstrated its good electrofluidic performance and operation.

## 2. Materials and Methods 

### 2.1. Chemicals and Materials

Silver nanoparticle (AgNP) ink was purchased from Advance Nano Products Co., Ltd (DGP 40LT-15C, Sejong, Korea). The surface tension and the average particle size were 22 mN/m and less than 50 nm, respectively. For the ink cartridge and the housing barrel of the ballpoint pen, we used the cartridge from a ball pen (ball diameter of 1.0 mm, Zebra, Tokyo, Japan) and the barrel from a permanent marker (Monami, Yongin-si, Korea), respectively. Inkjet photo paper (C13S042187, Epson, Tokyo, Japan) was selected for the printing substrate because it is glossy and thermally stable up to a temperature of 200 °C. A digital plotter (Cricut Explore Air, Provo Craft & Novelty, Inc., South Jordan, UT, USA) with software (Cricut Design Space, Ver. 3) was employed for the printing. The ballpoint pen used for the printing with a digital plotter was prepared as reported previously [20]. After the original ink had been removed, the empty cartridge was sonicated in an ethanol bath for a day, after which it was cleaned with water. The cleaned cartridge was then filled by pipetting with 80 μL of AgNP ink. A plastic film, which is used to wrap food in a home kitchen (thickness of ~17 μm, Cleanwrap, Seoul, Korea) and is made of flexible, stretchable linear low-density polyethylene (LLDPE) was used. For both the lubricant and the adhesive filler, dielectric silicone oil with a dynamic viscosity of 10 cP (Sigma-Aldrich, St. Louis, MO, USA) was used.

### 2.2. Printing of Electrode Arrays for Paper-Based DMF Chip

In the patterning of electrodes for the paper-based DMF chip, we used AgNP ink, which is a highly conductive material, a ballpoint pen, and a digital plotter. The electrode arrays for the paper-based DMF chips were designed using computer-aided design software (Adobe Illustrator CC 2015, Adobe, San Jose, CA, USA) and then exported to an AutoCAD Interchange file (*.DXF). Before the printing, the prepared ballpoint pen was inserted into clamp A of the digital plotter. Then the design file was uploaded to an online design space of the digital plotter and set as the writing function. We set the printing file as the writing function (Clamp A) with a printing speed of 5 cm/s. The printing speed was fixed at 4.6 cm/s for a horizontal or vertical line and at 5.9 cm/s for a 45°-tilted line. After the printing, the AgNP electrode arrays were annealed at 170 °C for 30 min to reduce electrical resistance. We characterized the printed patterns by using a field emission scanning electron microscope (FE-SEM) (JSM-7100F, JEOL, Pleasanton, CA, USA).

### 2.3. Preparation of Thin Dielectric Film for Paper-Based DMF Chip

The dielectric layer for the paper-based DMF chip was prepared using a commercial pre-made plastic wrap (17 μm) made of LLDPE. It was prepared by fixing it to an adhesive plastic frame to make it flat. We applied silicone oil (20 μL) to cover the entire surface of the LLDP-dielectric layer to reduce the layer’s surface friction during the movement of a droplet on the surface. We applied a thin layer of silicone oil to the printed electrodes and to the substrate to ensure that the LLDPE-dielectric layer bonded to the printed electrodes and the substrate with no air bubbles at the interface. The surface properties of the LLDPE-dielectric film were observed by using atomic force microscopy (AFM) (NanoStation, Pucotech, Seoul, Korea) and Fourier-transform infrared spectroscopy (FT-IR) (Agilent Technologies, Cray 640 FTIR, Santa Clara, CA, USA). The contact angles (CA) of droplets were measured by using a freeware program (ImageJ, 1.51p, National Institutes of Health (NIH), Bethesda, MD, USA) on images of water droplets captured by using a contact angle analyzer (Phx 300, Image XP 5.6U, SEO, Seoul, Korea). The leakage of electrical current was measured by using a source meter (Keithley, 2400, Keithley Instruments, Solon, OH, USA).

## 3. Results

### 3.1. Electrode Arrays of the Paper-Based DMF Chip

**Figure 1:**
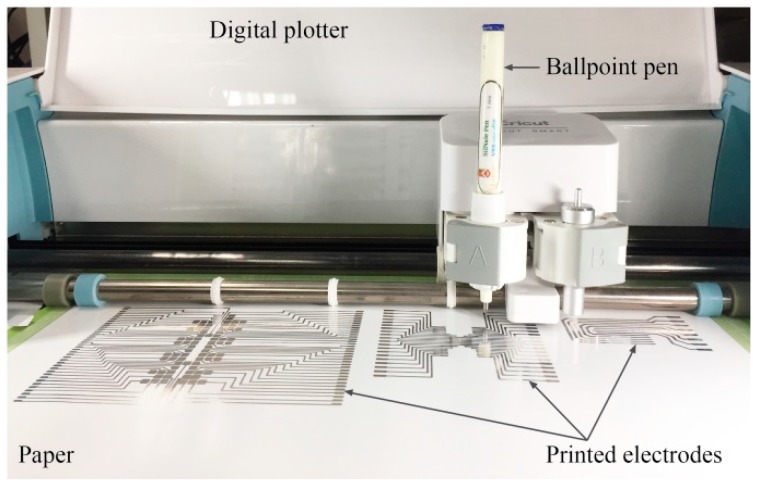
Printing setup for patterning electrode arrays on paper for the fabrication of a digital microfluidic (DMF) chip by using a ballpoint pen and a digital plotter.

In the fabrication of the DMF chip, the electrode size and shape must be carefully designed and depend on the sample volume and whether the system is closed or open. Obtaining a proper method for printing a customized electrode is crucial. To that end, we explored a simple printing method using a customized ballpoint pen [20,21,22] and a type of direct contact printing in which we simply replaced the pigment ink in the stylus with conductive AgNP ink. After the ballpoint pen had been prepared, we inserted it into a pen holder of the plotter for printing in a programmable manner [20]. The contact pressure and the lateral movement of the ballpoint pen controlled by the digital plotter allowed the deposition gap of the ballpoint pen to open, and rotated the ballpoint of the ballpoint pen. As a result, AgNP ink was deposited on the printing substrate. Figure 1 shows the printing setup for patterning electrode arrays for a paper-based DMF by using a ballpoint pen and a plotter. With the printing system, a layman can print a customized design on paper in minutes without the need for expensive equipment (Video S1).

**Figure 2:**
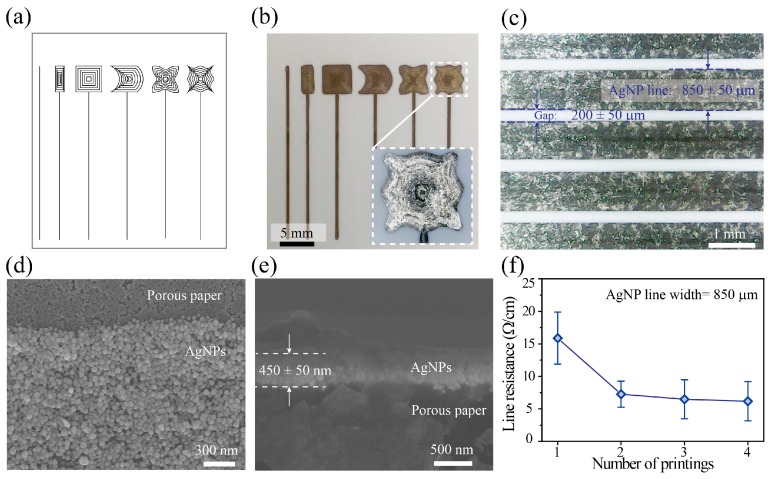
Printing silver nanoparticle (AgNP) electrodes on paper. (**a**) Electrode designs with various sizes and shapes, (**b**) electrodes printed with the designs in (**a**) and (inset) an enlargement of one design, and (**c**) printed AgNP lines. SEM images of the printed pattern: (**d**) top view and (**e**) cross-sectional view. (**f**) Surface electrical resistance of the printed AgNP line versus the number of printings.

In Figure 2a,b, we demonstrate the printing on paper of AgNP electrodes with the various sizes and shapes that are commonly used in DMF chips and with the desired designs. However, if a large electrode is to be printed, the printed electrode has to be designed as a group of lines [20]. The printed electrodes showed clean edges and good resolution, and by printing the AgNP electrodes using a 1-mm ballpoint-pen diameter, we were able to generate in a single printing an electrode with a minimum width and gap of 850 ± 50 μm and 200 ± 50 μm, respectively (Figure 2c). The SEM image shows that the printed electrode contained a high density of AgNPs connected to their neighbors (Figure 2d). Moreover, the thickness of the printed electrode (single printing) was 450 ± 50 nm, which is suitable for DMF use (Figure 2e).

Because the printed AgNPs are, manifestly, neither strongly metallic bonded nor perfectly crystallized, but rather a grouped domain of aggregated nanoparticle grains, in order to enhance the conductivity of the AgNP film and to make it more tightly bonded, we thermally annealed the printed AgNPs at 170 °C for 30 min. This low-temperature annealing was selected in consideration of the limited thermal resistance of the photo paper (210 °C) (Figure 2c). With our setup, the electrical resistance of the AgNP pattern was about 16 Ω/cm for one printing, was reduced to about 7 Ω/cm for two printings and was slightly less for three and four printings (Figure 2f). The slight reduction in electrical resistance for three and four printings is because the first printed layer can be squeezed by a ballpoint pen during the printing of a next printed layer. This is a limitation of the contact printing technique in the printing of multi-layer patterns. Although increasing the number of printings increased the electrical conductivity of the printed pattern, with our printing setup, one printing was sufficient to generate a highly conductive electrode for a DMF chip. These results clearly show that our printing method has the ability to fabricate electrodes in arbitrary patterns. Furthermore, the deposition of a thin electrode is interesting because such an electrode has definite advantages in many applications, including DMF chips.

### 3.2. Dielectric Layer of the Paper-Based DMF Chip

Our easy printing of conductive materials to obtain precisely patterned electrodes meets the challenge of being a simple coating process for achieving highly hydrophobic dielectric films and offers an affordable way to fabricate paper-based DMF chips. The intervening dielectric thin film allows the use of high voltages for inducing a stronger electrowetting force so that the shape of the drop can be sufficiently deformed, and the drop can eventually move [3]. However, since Berge introduced an excellent dielectric material, parylene-C, which has a very high electrical breakdown voltage (200 V/μm), new attempts to obtain alternative dielectrics have been surprisingly rare, except for a few materials, such as Teflon, SU-8, CYTOP, and polydimethylsiloxane (PDMS) [3,19,23,24,25,26]. Even worse, most thin-film deposition methods, such as CVD for parylene-C and spin-coating for Teflon, require heavy instruments, including gas- and temperature-control systems on a lab-scale, which is a severe obstacle to the affordable fabrication of DMF chips.

**Figure 3:**
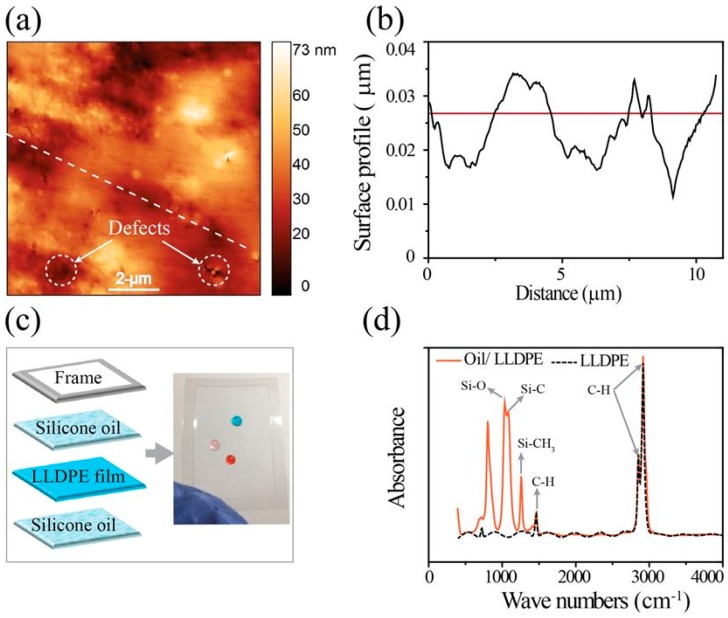
Surface morphology of the commercial LLDPE film. (**a**) Atomic force microscopy (AFM) image of the film and (**b**) representative surface profile extracted from (a) at the dashed line. (**c**) Schematic structure of a dielectric film prepared for the paper-based DMF chip and a photograph of the prepared dielectric film with three colorful drops on it. (**d**) FT-IR spectrum of a LLDPE-dielectric film with and without a silicone oil coated.

**Figure 4:**
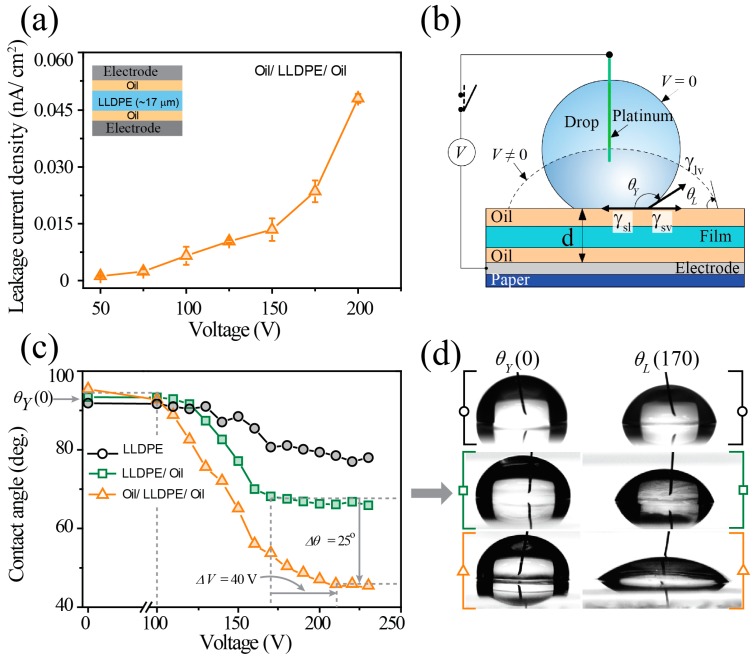
(**a**) Leakage current density across the LLDPE-dielectric film and its schematic measurement (inset). Characterization of the electrowetting on dielectric (EWOD) experiment on the LLDPE-dielectric film: (**b**) EWOD setup, and (**c**,**d**) the electro-spreading of the drop under applied voltages.

Because of the above factors, we explored a new approach, the hand-wrapping approach, to fabricate a dielectric film for a paper-based DMF chip by using a commercially available plastic film made of LLDPE. Originally, this method was developed by Gaudi Labs in 2017 and was then made available to the public [24]. Even though commercial, pre-made LLDPE wrap is low-cost, the pristine wrap cannot be used for the dielectric layer of a DMF chip without treatment. AFM data showed that the surface of the pre-made LLDPE film contained some defects (Figure 3a,b). These defects may allow electrical current leakage during the operation of a DMF chip under high voltage. Low hydrophobicity (contact angle: ~93°) is another drawback of the pre-made LLDPE film; this causes the surface friction to increase, thereby impeding the movement of drops during DMF chip operation. To overcome these limitations, we applied a thin layer of silicone oil on the LLDPE-dielectric film in order to make the surface slippery, thus reducing the surface friction [27,28,29] (Figure 3c). For ease of handling and the avoidance of wrinkles, we used an adhesive plastic frame, to which the LLDPE-dielectric film was fixed to make it flat (Figure 3c). With the FT-IR spectrum, we confirmed the presence of a thin layer of silicone oil on the LLDPE-dielectric film (Figure 4d). We believe that covering the LLDPE film with a thin layer of silicone oil seals the surface defects on the surface of pre-made LLDPE, which should minimize the leakage of electrical current during high-voltage DMF chip operation. We observed the leakage current across the LLDPE-dielectric layer treated with silicone oil by applying various voltages from 50 V to 200 V (Figure 4a). The leakage current density dramatically increased when 50 V to 175 V were applied, and rapidly increased when a higher voltage was applied. However, this low leakage of electrical current can be ignored if the operation voltage is lower than 200 V. With our method, we were able to rapidly prepare and apply a dielectric layer for our fabricated paper-based DMF chip without the need for any special tools or devices. 

### 3.3. Droplet Actuation on the Paper-Based DMF Chip

For a sessile drop on the LLDPE-wrapped electrodes, we measured the CA as a function of the applied voltage (Figure 4b) so as to investigate the dielectric properties of the LLDPE-dielectric film. As shown in Figure 4c, the initial CA at no voltage was greater than 90°, indicating that the hydrophobicity of the film was sufficient for EWOD applications (black, Figure 4c). However, the CA response to changes in the applied voltage was irregular, not smooth, the so-called CA hysteresis [3], mostly due to the existence of an air gap under the LLDPE-dielectric film. In order to remove the air gap, we coated the bottom side of the LLDPE-dielectric film with a dielectric liquid sealant, silicone oil, to a thickness of roughly 200 nm [3,14,24]. After this sealant treatment, we obtained a much-improved CA response (green in Figure 4c), and the variation in the CA with applied voltage was small, being only *Δθ* = 93 − 68 = 25° for *ΔV* = 100 − 170 = −70 Vdc. Of course, we ignored both the initial inert wetting region at low voltages < 100 Vdc (leftmost dashed line in Figure 4c), and the saturated CA region at high voltages > 170 Vdc (middle dashed line in Figure 4c). To increase the differential CA, *Δθ*(*V*), we also coated the top of the wrapped film with silicone oil to lubricate the surface. As a result, *Δθ*(*V*) was doubled from 25° to 50° for *ΔV* = −40 Vdc (orange in Figure 4c). Figure 4d shows representative images of sessile drops wetted on the surface of the LLDPE-dielectric films at zero and 170 V taken from Figure 4c. 

According to the Young-Lippmann equation describing the relationship between the Young and the Lippmann contact angles, *θ_Y_*(0) and *θ_L_*(*V*), for the equilibrium states of the drop,

(1) $$\cos\theta_{L}(V)=\cos\theta_{Y}(0)+\frac{1}{2\gamma_{lv}}CV^{2}$$

where *γ_lv_* is the interfacial tension between the liquid and the vapor. A higher voltage is needed to compensate for the excessive thickness of the plastic wrapped film, 17 μm, because of the capacitance *C* = *εA*/*d*, where *A*, *d* and *ε* are the area, thickness, and permittivity of the capacitor [3,23,30]. Fortunately, however, due to the quadratic dependence on the voltage, the required voltage for our excessively thickly wrapped film was still low in the range of less than 210 Vdc (rightmost dashed line in Figure 4c), which can be provided by a small-size electronic power supply. If this were not the case, electric breakdown might occur because the required voltage for a 17-μm thickness would be 425 V due to the relatively low dielectric strength of 25 V/μm of the LLDPE-dielectric film. 

### 3.4. Operation of the Paper-Based DMF Chip

**Figure 5:**
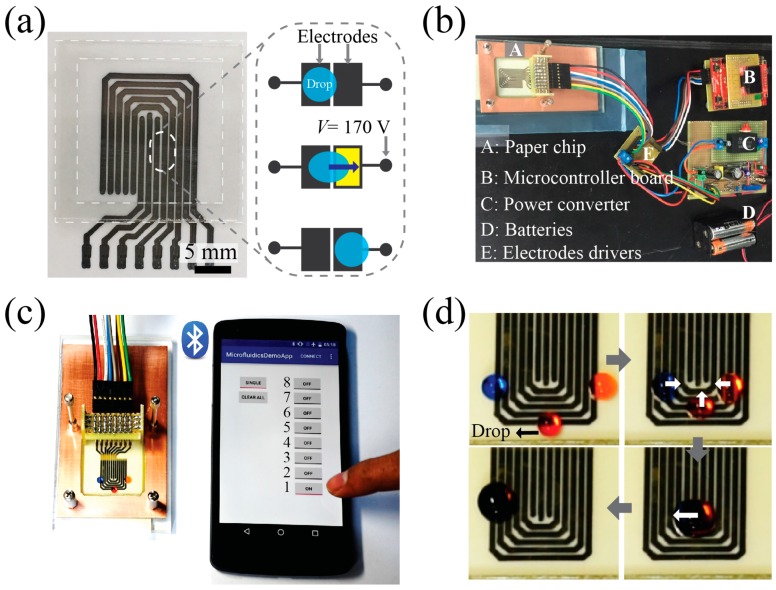
Demonstration of drop actuation on an affordable paper-DMF chip: (**a**) a printed paper chip and (inset) drop actuation scheme and (**b**) the chip connected to a power switching device. (**c**) A smartphone App being run through wireless Bluetooth, and (**d**) three digital drops initially at rest being simultaneously transported (top) to merge into one drop at the center and then being transported to the left (bottom).

Finally, using the same multilayer configuration for the EWOD experiment (Figure 4b), except for replacing the single electrode with a trail-patterned array of electrodes, we fabricated a paper-based DMF chip easily, rapidly and affordably. The paper-based DMF chip (area 5 cm × 5 cm) demonstrated in Figure 5a costs less than USD 1 to make with a setup that costs approximately USD 241. Because a high voltage (*V*_dc_ > 170 V) caused the drop’s spread to reach sub-maximal CA saturation, we operated our paper-based DMF chip at a voltage of 170 V. With the applied voltage, the electrode arrays (width: 800 μm, gap: 400 μm) could move a 5-μL drop along the activated electrode (yellow mark, inset of Figure 5a). The drop was sufficiently deformed and touched the adjacent electrode, eventually moving toward the activated electrode. The drop’s movement across the planar electrodes can be attributed to the large differential variation of CA, *Δθ* = 50 °C (orange, Figure 4c) because the actuation EWOD force is proportional to *Δθ* according to Furmidge’s equation [30,31]. Similarly, we successfully demonstrated digital fluidic manipulation along the trail-patterned electrodes.

A 15-μL drop was formed by transporting and merging three 5-μL drops along different paths after the paper-based DMF chip had been connected to a custom designed control system (Figure 5d, Video S2). The control system consists of a hardware prototype and a smartphone, as shown in Figure 5b,c. The smartphone runs a custom-developed app that can send commands over a Bluetooth link to the hardware prototype. The hardware prototype is based on the Stelaris LM4F120 Launchpad (Texas Instruments, Dallas, TX, USA) development board, which is interfaced with a Bluetooth module, a display, a custom-developed isolated flyback boost converter, and an electrode driver board based on a commercially available integrated circuit (HV2201, Microchip, Chandler, AZ, USA). The control system design is similar to that in our previous reports [8,32]. Our paper-based DMF chip can be used more than 100 times without damage. In addition, it is a benefit of the design that the dielectric layer can be substituted, which is different from the parylene coatings that are attached to the surface.

## 5. Conclusions

In conclusion, two affordable methods for generating both conductive electrode arrays and dielectric layers for use in the fabrication of paper-based DMF chips were introduced. The conductive electrode arrays for the DMF chips were printed according to a program on paper by using the prepared ballpoint pen and a digital plotter. In the preparation of the dielectric layer for the paper-based DMF chip, we used commercial food wrap made of LLDPE. We framed and treated the LLDPE-dielectric film with a thin layer of silicone oil on both surfaces (front and back). The treatment provided a slippery surface and covered the surface defects in the LLDPE-dielectric film, thereby preventing the leakage of current during chip operation. We investigated the properties of the LLDPE-dielectric film, and the results showed that the film sufficiently satisfied the dielectric requirements for a multilayer DMF chip. This approach holds much promise as a simple, easy, and rapid way to fabricate paper-based DMF chips affordably.

## Supplementary Materials

The following are available online at http://www.mdpi.com/2072-666X/10/2/109/s1, Video S1: Printing on paper the conductive electrode array for the DMP, Video S2: Operation of the DMF with a smartphone App.
